# Variability of Prognostic Results Based on Biological Parameters in Sickle Cell Disease Cohort Studies in Children: What Should Clinicians Know?

**DOI:** 10.3390/children8020143

**Published:** 2021-02-13

**Authors:** Julie Sommet, Enora Le Roux, Bérengère Koehl, Zinedine Haouari, Damir Mohamed, André Baruchel, Malika Benkerrou, Corinne Alberti

**Affiliations:** 1 AP-HP, Hôpital Robert-Debré, Unité d’épidémiologie clinique, Inserm, CIC 1426, 48 bd Serurier, F-75019 Paris, France; enora.leroux2@aphp.fr (E.L.R.); corinne.alberti@aphp.fr (C.A.); 2 Université de Paris, UMR 1123, ECEVE, 10 av de Verdun, F-75010 Paris, France; malika.benkerrou@aphp.fr; 3 AP-HP, Hôpital Robert-Debré, USC de chirurgie pédiatrique, 48 bd Serurier, F-75019 Paris, France; 4 AP-HP, Hôpital Robert-Debré, Unité d’Epidémiologie Clinique, 48 bd Serurier, F-75019 Paris, France; damir.mohamed@gmail.com; 5 AP-HP, Hôpital Robert-Debré, Centre de Référence de la Drépanocytose, Service d’Hématologie, 48 bd Serurier, F-75019 Paris, France; berengere.koehl@aphp.fr (B.K.); zinedine.haouari@aphp.fr (Z.H.); 6 Université de Paris, INSERM UMR1134, Institut National de la Transfusion Sanguine, (INTS),6, rue Alexandre Cabanel, F-75015 Paris, France; 7 AP-HP, Hôpital Robert-Debré, Service d’Hématologie, 48 bd Serurier, F-75019 Paris, France; andre.baruchel@aphp.fr; 8 Université de Paris, Equipe d’accueil 3518, Institut Universitaire d’Hématologie, Hôpital Saint Louis, 1 Av Claude-Vellefaux, F-75010 Paris, France

**Keywords:** sickle cell disease, cohort, biological parameter

## Abstract

Background: Many pediatric studies describe the association between biological parameters (BP) and severity of sickle cell disease (SCD) using different methods to collect or to analyze BP. This article assesses the methods used for collection and subsequent statistical analysis of BP, and how these impact prognostic results in SCD children cohort studies. Methods: Firstly, we identified the collection and statistical methods used in published SCD cohort studies. Secondly, these methods were applied to our cohort of 375 SCD children, to evaluate the association of BP with cerebral vasculopathy (CV). Results: In 16 cohort studies, BP were collected either once or several times during follow-up. The identified methods in the statistical analysis were: (1) one baseline value per patient (2) last known value; (3) mean of all values; (4) modelling of all values in a two-stage approach. Applying these four different statistical methods to our cohort, the results and interpretation of the association between BP and CV were different depending on the method used. Conclusion: The BP prognostic value depends on the chosen statistical analysis method. Appropriate statistical analyses of prognostic factors in cohort studies should be considered and should enable valuable and reproducible conclusions.

## 1. Introduction

Although all patients with sickle cell disease (SCD) share a specific genotype mutation, clinical variability in the pattern and severity of disease manifestations is broad and unpredictable: for example, some patients develop serious complications such as cerebral vasculopathy (CV) while others do not. Physicians and researchers have sought to identify the factors that explain the phenotypic variability of sickle cell disease (SCD) and predict its clinical course in patients. Over recent decades, more than fifty prognostic studies have been published, especially in children, seeking to understand the factors associated with increased morbidity and mortality. Several studies have identified prognostic variables [1] potentially associated with increased disease severity, including fetal hemoglobin level (HbF) [2,3], leukocyte count [4,5], reticulocyte count [6] or hemoglobin level [7,8].

However, there is variability in the results in published studies. For example, HbF was shown to be protective against cerebral vasculopathy (CV) in one study [9], while others failed to demonstrate this [6,7]. Decreased hemoglobin was found to be a risk factor for CV in two studies [10,11] but not in other studies [6,9]. An increased reticulocyte count was found to be a risk factor for CV in four studies [6,9,10,11], but not in the Hankins study [12]. These examples highlight the difficulties to predict SCD phenotype even if the differences in the results may be due not only to the available sample size but also to differences in the methods used to analyze the biological parameters.

Blood levels of these biological parameters do indeed change over time, particularly in children. First, baseline levels vary physiologically depending on age. For example, the rate of HbF usually decreases by 60% at 3 months of age, down to 25% at one year of age [9]. In addition, blood levels may vary as a result of the disease course or the occurrence of complications, or during some treatments.

The aim of our study was (i) to assess how the biological parameters are collected and used in statistical analyses in cohort studies in the literature, (ii) to study whether the methodology adopted has an impact on the conclusions drawn with regard to the possible association between biological parameters and clinical SCD events in the pediatric context. In particular, we investigated the impact of statistical strategies by comparing them when studying the relationship between biological parameters and CV in our pediatric cohort of sickle cell patients followed at the Robert Debré University Hospital.

## 2. Methods

In the first step, a literature search was performed to review how the biological parameters were collected and used in statistical analyses in published cohort studies in SCD. The second step was to perform different statistical analyses on our cohort dataset of SCD patients in order to assess the impact of the methodological strategy on the results for this given cohort and therefore, potentially, on the overall conclusions of the studies.

### 2.1. Literature Search

We defined a SCD cohort study as a longitudinal study including a group of SCD children with defined characteristics (age, sex, genotype for example), who were followed up to determine the incidence of all causes of death or some other SCD related outcomes. This implied that biological data and clinical outcomes were collected repeatedly over time during the follow-up. We focused on cohort studies because cross-sectional or case-control studies are based on a snapshot, and therefore only one measurement of biological parameters is generally observed, whereas several values over time are usually collected in cohort studies.

We selected cohort studies conducted over a 10-year period in children from 0 to 18 years of age, which evaluated the association between the main biological parameters linked to SCD, i.e., hemoglobin and/or fetal hemoglobin and/or reticulocyte count, and/or leukocyte count, and a clinical event linked to SCD. Our aim was to describe the statistical methods used in studying such relationships. Selected studies were those published in English in the four leading journals in terms of impact factor according to Journal Citation Reports, 2016 in the following categories: “paediatric” or “hematology” or “general journals”. We did not include conference reports or short communications, commentaries or letters to editors, as the methods are usually poorly reported in such articles.

Two researchers (EL, JS) independently screened the retrieved records and selected all studies that fulfilled the eligibility criteria. The same researchers (EL, JS) independently screened the titles and abstracts to identify relevant studies. Full texts were read when abstracts met inclusion criteria or when abstracts were not sufficiently clear to determine eligibility. Full-text screening was completed independently. All disagreements were discussed and resolved by consensus.

The researchers (EL, JS) independently extracted data from each relevant study using a standardized computerized data collection form developed for the study. Disagreements were resolved by discussion and consensus.

The following information was extracted: authors, article title, publication year, journal, country, setting, number of patients included, patients’ ages at study inclusion, SCD genotypes, study design and SCD clinical events studied. We also reported the data collection timeframe and the statistical methodology used. When information was not available, investigators were contacted by the researchers to request additional information.

### 2.2. Illustration of the Impact on Results of the Methods Selected

To illustrate the impact of the methods by which prognostic variables are collected and taken into account in statistical analyses, we applied them to our SCD pediatric cohort dataset, collected prospectively from the National Paediatric Sickle Cell Reference Centre at the Robert Debré University Hospital in Paris, France.

#### 2.2.1. Study Population

The studied outcome was cerebral vasculopathy (CV), an important complication of sickle cell disease in children. The cohort was previously described in Sommet et al. [9]. In summary, the cohort included 375 patients, 363 of them with homozygous HbS and 12 with heterozygous sickle cell/beta°-thalassaemia. Median follow-up was 6.8 years (min: 0.6; max: 17.9) (2677 patient-years). In this cohort, biological data and concurrent clinical status were recorded prospectively at 3, 6 and 12 months of age and yearly thereafter. During the follow-up, each biological variable was measured a median of 6 times (min: 1; max: 16).

#### 2.2.2. Statistical Methodology

Biological parameters were assessed as risk factors for CV using a Cox survival multivariable model. Time-to-CV was calculated from the date of birth to the date of diagnosis of CV. Patients who remained CV-free at their last follow-up before 31 December 2010 were right-censored at this date. Patients not seen during the 12 months prior to 31 December 2010 were considered lost to follow-up and censored at their last follow-up. We used the seven available biological variables evaluated as risk factors in our cohort study: reticulocyte count, platelet count, neutrophil count, mean corpuscular volume, leukocyte count, hemoglobin level and HbF level.

To assess the prognostic value of biological parameters in this model, we conducted our analyses according to the identified methods retrieved from the literature review: (1) one value per patient at inclusion in the study (from 1 to 12 months of age); (2) last known value, whatever the age at the last follow-up; (3) mean of non-censored values per patient; (4) modelization of all non-censored values in a two-stage approach as previously described [13,14]. This last method enables the entire evolution over time of biological data to be taken into account. In the first stage, all observed values were modelled using a linear mixed model. Then, in the second stage, to incorporate variation of each biological parameter (for example, HbF decrease in the first year of life), estimates for each subject were entered into the survival model (or Cox model).

We applied these four methods to all available biological parameters measured several times during the follow-up: reticulocyte count, platelet count, neutrophil count, mean corpuscular volume, leukocyte count, hemoglobin level and HbF. We did not include clinical variables because a formal study of risk factors for cerebral vasculopathy was the aim of our previously published study [9]

For each analysis, we included all biological variables in the final multivariable model with stepwise selection classically applied in multivariable analyses. Use of a stepwise selection procedure allows for selection of statistically significant variables only at the last step of the procedure. The alpha level was set at 0.05 for all analyses. The results are presented as the hazard ratio (HR) with 95% confidence interval (95% CI). It was performed with SAS 9.4 (Cary, NC, USA).

## 3. Results

### 3.1. Literature Search

The electronic literature search retrieved a total of 571 references, from which 65 were selected based on the titles and abstracts. Sixteen studies met the inclusion criteria and were included in the review.

Characteristics of the studies included are shown in Table 1. The age at entry to the cohorts varied from birth to 17 years. Details for each study are provided in the Supplementary Table S1.

### 3.2. Collection and Statistical Analyses of Biological Parameter in Cohort Studies

The biological parameters collected in the 16 studies and evaluated as prognostic factors were the hemoglobin level (*n* = 16), the reticulocyte count (*n* = 14), leukocyte count (*n* = 14) and the HbF (*n* = 11). The methods used to collect biological parameters in the 16 SCD cohort studies are presented in Table 2. The parameters were collected:

− either once during the follow-up: either at a fixed age range (which depended on the study), or at the last follow-up;
− or several times: either all values during the follow-up or at a fixed range of ages.

It should be noted that several methods of data collection were used in a single study, depending on the parameter.

The statistical methods used also differ from one study to another. In some cases only one value was used, which could be either the value at inclusion in the cohort or the last-known value. In others studies, the mean of all non-censored values per patient was used in the statistical analysis and in one study all non-censored values were modelled in a two-stage approach.

### 3.3. Illustration of the Impact on Results of the Methods Selected

**Figure 1:**
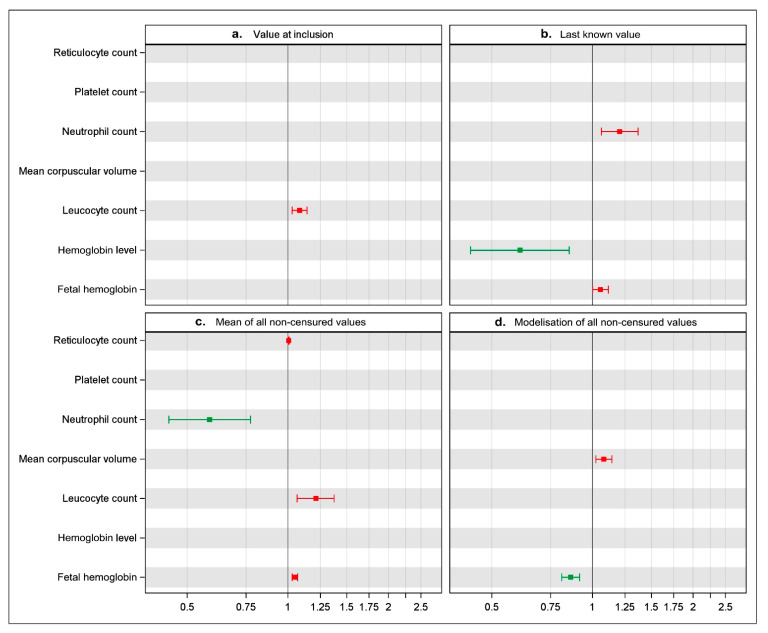
Illustration of results obtained according to the four methods used to enter the variables in the cox model studying the relationship between the biological parameters and the occurrence of cerebral vasculopathy.

In our own prospective cohort of newborn SCD patients, we selected seven biological parameters likely to be prognostic in the occurrence of cerebral vasculopathy in SCD children: hemoglobin level, reticulocytes count, mean corpuscular volume, platelet count, leukocyte count, neutrophil count, and fetal hemoglobin. We analyzed the association between these parameters and the occurrence of CV by using successively the four methods previously described (value at inclusion, last known value, mean of all non-censured values, and modelization of all non-censured values). The results of these analyses at the last step of the stepwise multivariable model are shown in Figure 1.

Each panel represents one of the four methods used in multivariable analysis, i.e., a: one value at inclusion in the study; b: last known value; c: mean of all non-censored values per patient; d: modelization of all non-censored values in a two-stage approach. Hazard ratios with 95% confidence intervals for each laboratory parameter are represented by square and horizontal lines, respectively. There was a significant association between the laboratory parameter and CV when the line does not cross the vertical line corresponding to one in abscissa: as a protective factor (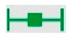), in green, when the square is to the left of the line and as a risk factor (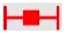) in red when it is to the right. Stepwise selection is a statistical method in which statistically significant prognostic variables are selected using an automated procedure to build a multivariable model. When this procedure is applied, only variables with a 5% level of significance are captured.

Even if the same variables were used in each model, their values were different depending on the method used.

− If we considered the single value at the inclusion of the study (panel a), we found that only the leukocyte count was associated with a higher risk of CV (HR 1.11 (1.03–1.15)). The six other biological parameters were not associated with the occurrence of CV.
− If we considered the single last known value (panel b), we found that only the neutrophil count and the HbF level were associated with a higher risk of CV (respectively, (HR 1.20 (1.07–1.35) and 1.13 (1.10–1.19))) whereas the hemoglobin level was inversely associated with the risk of CV (HR 0.62 (0.39–0.86)). The four other biological parameters were not found to be significantly associated with the occurrence of CV.
− If we considered the mean of all non-censured values (panel c), we found that the neutrophil count was inversely associated with the occurrence of CV (HR 0.59 (0.27–0.79)), whereas the reticulocyte count, the leukocyte count and the HbF level were associated with a higher risk of CV (respectively, (HR 1.02 (1.01–1.03)), (HR 1.22 (1.07–1.36)) and (HR 1.08 (1.04–1.10))). The three other biological parameters were not found to be significantly associated with the occurrence of CV.
− Last, if we performed a modelization of all non-censured values (panel d), we found this time the mean corpuscular volume to be associated with the occurrence of CV (HR 1.08 (1.02–1.15)), whereas the HbF level was inversely associated with risk of CV (HR 0.87 (0.82–0.92)). The five other biological parameters were not found to be significantly correlated with the occurrence of CV.

Overall, the final number of significant parameters can differ from one to four, depending on the statistical method used. Furthermore, one biological parameter can be found to be significant with a method and non-significant with another. Finally, the same biological parameter can be described as protective when analyzed with one of the methods, and a risk factor when analyzed with another.

## 4. Discussion

Hematological parameters are often described as prognostic factors in sickle cell cohort studies, but there is variability in their analysis and interpretation in the literature. Therefore, we first listed the different methods used to collect them and how they were used in the statistical methodology in this field. Next, we investigated how the way of using biological parameters in a Cox model, studying as an example the relationship between these parameters and the occurrence of a severe and unpredictable complication in SCD patients, the CV. In this manner, we highlighted that depending on the method used, different conclusions on the association between biological parameters and CV are achieved.

This result raises questions because the biological parameters are prognostic markers in SCD children but are also the target of a main treatment, hydroxycarbamide. Regarding our illustration, fetal hemoglobin is a protective factor for CV when using the trajectory along age by the two-stage approach (panel d) while it is a risk factor when using the last value (panel b) or the mean (panel c) of the values. Thus, the message concerning the prognostic value of the biological parameter and the use for clinical practice may not be clear. For example, in the study conducted by Curtis et al. [15], which enrolled 359 patients with sickle cell anemia, lower baseline levels of HbF were associated with increased mortality, whereas, in the same database, change in percentage of HbF was not a significant risk of increased mortality. Indeed, the handling of biological parameters in statistical analyses influences the conclusions concerning their association with the SCD complications. This could explain in part the variability of the results in the published studies. Once again, HbF was shown to be protective against CV in one study [9] while other studies failed to demonstrate this [6,7]. However, the protective role has been well established for other SCD complications [16]. Higher HbF levels are known to prolong survival [2], [16], and to protect against vaso-occlusive crisis and acute chest syndrome [3,17,18] and leg ulcers [19,20].

Longitudinal design is particularly important in children studies since hematological biological parameters change with age (or over time) and HbF interpretation in the literature clearly illustrates this. In order to rigorously assess the prognostic or non-prognostic status of the HbF level in children between 18 months and 5 years of age, which is the period of onset of this complication, it is important to bear in mind that HbF levels decrease by approximately 25% to 15% over this age range. Blood hemoglobin levels also vary depending on age, with very high levels in newborns, followed by a gradual decrease, with the lowest levels observed between 1 and 6 months of age, followed by a gradual increase and then stabilization at puberty [21]. The variations in hematological parameters depending on age [21,22] must be taken into account, especially because of the absence of standards according to age and sex in the SCD children population, whereas pediatric ranges are usually used in other fields, e.g., growth for example [23].

Thus, statistical methods that reduce the information from longitudinal data to a single value can lead to biased conclusions. Two patients can have similar outcomes at a point in time but they can have very different evolution if we consider a longer period of time. Likewise, the evolution over time cannot be reflected by the average of all values, which masks extreme values and change over time; in any case appropriate models exist for taking into account the longitudinal evolution of data instead of solely one value. However, it is true that using longitudinal follow-up raises others questions. It is costly and can lead to a substantial amount of missing data, particularly if measurements are repeatedly taken over time. In the two-stage approach, measurements are modelled over time with linear mixed models, making it possible to estimate their trajectory in the presence of missing data. Cohort studies are robust designs for evaluating the association between measured factors and the occurrence of health events. The major cohorts used in health fields are the result of multidisciplinary collaboration, e.g., between clinicians, sociologists, biologists, epidemiologists and statisticians. To our knowledge, this is the first work that explores how laboratory parameters are used to predict outcome in SCD studies in children. A limitation of our work is that we did not include clinical variables in the statistical illustration. This is because we did not want to produce a prognostic study but to highlight the importance of statistical analysis choices on the accuracy of the results. However, our work raises fundamental questions about methodological approaches relevant for clinician researchers. Even if they can request statistician support, they need to be aware of the importance of the choice of methods in producing good results and in their interpretation.

The two-stage approach has been adopted in multiple areas of clinical research, including HIV infection [13,24,25,26], cancer [27], cardiovascular diseases [28], and kidney transplantation studies [29,30]. In the field of SCD, a two-stage model was used for the first time only recently [9]. However, there is still a margin for improvement with the use of joint models. Standardization of practices and methods in prognostic studies in SCD children would help to avoid so-called vibration of effects, whereby results can differ, i.e., vibrate over a wide possible range, depending on how the analysis was conducted [31]. Standardization and homogenization of methods could lead to more transparent reporting in these studies and enhance the reliability of published data [32]. Large consortia and collaborations would allow investigators to use a common language for clinical definitions, biological parameters collection and statistical analysis.

## 5. Conclusions

Our work revealed heterogeneity among studies that investigate factors associated with clinical events in SCD, in terms of analysis methods and results. We showed that using different methods on the same data set can lead to different conclusions as to the prognostic value of the biological parameters in predicting SCD morbidity, illustrating how important it is to apply appropriate statistical and data collection methods. We suggest that assessing prognostic values and monitoring their changes over time may be an improved way of assessing SCD prognosis. This may have important implications in disease stratification for personalized therapeutic interventions. Collaborative work should be initiated on methodologies, including clinicians and statisticians, to obtain the most reliable results and enhance feasibility in terms of collection, in the context of observational studies. It seems reasonable to strive to move towards greater standardization of practices, especially in pediatric cohort studies, in order to limit variability and to strengthen the reliability of results, so as to allow for comparisons across studies, and to ensure transparent reporting.

## Figures and Tables

**Table 1:** Characteristics of the 16 sickle cell disease (SCD) cohort studies found by the literature search study characteristics.

	*N* = 16
**Region**	
North America	8
Europe	7
Africa	1
**SCD clinical event studied**	
Neurological complications *	10
Vaso-occlusive crises	2
Acute chest syndrome	1
Nutrition and growth	2
Pulmonary arterial hypertension	1
Alloimmunization	1
Retinopathy	1
Hemolysis	1
Mortality	1
**Number of included patients (*n* = 16)**	
Median (Q1; Q3)	184 (102; 376)
(Min-Max)	(24; 1041)
**Age at study inclusion (years) (*n* = 9)**	
Median (Q1; Q3)	2.3 (0.8; 11.8)
(Min-Max)	(0.3; 17.0)
**Follow-up in years (*n* = 10)**	
Median (Q1; Q3)	6.0 (2.0; 11.5)
(Min-Max)	(2.5; 6.7)

* Neurological complications are cerebral vasculopathy, stroke or silent infarct.

**Table 2:** Description of the methods used to collect and to analyze biological parameters in the 16 SCD cohort studies.

Laboratory Parameter	Hemoglobin		Reticulocyte Count		Leukocyte Count		Fetal Hemoglobin	
	*N*	%	*N*	%	*N*	%	*n*	%
**Methods used for data collection**	**16**		**14**		**14**		**11**	
*One value per patient*	*7*	*(44)*	*7*	*(50)*	*6*	*(43)*	*5*	*(46)*
At fixed age	7		6		6		4	
Last known value	0		1		0		1	
*Several values per patient*	*6*	*(37)*	*5*	*(36)*	*6*	*(43)*	*4*	*(36)*
All values during follow-up	3		3		3		2	
Values measured at fixed range of age	3		2		3		2	
*NR*	*3*	*(19)*	*2*	*(14)*	*2*	*(14)*	*2*	*(18)*
**Methods used for data modelling**	**15**		**13**		**12**		**11**	
One value per patient *	*8*	*(53)*	*8*	*(61)*	*5*	*(42)*	*5*	*(45)*
*Several values per patient*	*5*	*(33)*	*4*	*(31)*	*5*	*(42)*	*4*	*(36)*
							1	
*-* Values were summarized as means **	4		3		*4*		*3*	
- All values were modelled ***	1		1		*1*		*1*	
*NR*	*2*	*(13)*	*1*	*(8)*	*2*	*(16)*	*2*	*(18)*

*NR: Not Reported.* In the statistical model studying the relationship between the laboratory parameter and the event: * only one value was entered in the model. It could be either the value at inclusion in the cohort or the last-known value; ** the mean of all non-censored values per patient was entered in the model; *** all non-censored values were modelled in a two-stage approach as described in [13,14].

## Data Availability

Not applicable.

## Supplementary Materials

The following are available online at https://www.mdpi.com/2227-9067/8/2/143/s1, Table S1: Study characteristics (chronological order).
