# Supplementary material for: Variability of Prognostic Results Based on Biological Parameters in Sickle Cell Disease Cohort Studies in Children: What Should Clinicians Know?
Source: Children (Basel). 2021 Feb 13;8(2):143. doi: 10.3390/children8020143 (PMC7917793; doi:10.3390/children8020143)
Supplement: Supplementary file 1 [file children-08-00143-s001.pdf]

Table A1. Study characteristics (chronological order).

**References**	**[1]**	**[2]**	**[3]**	**[4]**	**[5]**	**[6]**	**[7]**	**[8]**
Publication year	2008	2008	2009	2010	2011	2011	2012	2012
**Study**								
-Region	Europe	North America	North America	Europe	Europe	Africa	North America	Europe
-SCD event studied	CV	CV	Silent infarct	PAH	CV	Growth Mortality	VOC	Acute chest syndrome VOC
**Population**								
-Inclusion number	373	274	65	37	217	1041	381	93
-SCD genotype	SS	SS/Sβ°	SS	SS/Sβ°/SC	SS/Sβ°	SS	SS	SS/SC
-Age at inclusion (years)	From 1 to 3	From 2 to 16	3.6 (means)	6.25 (means)	From 1 to 3	10.1 (means)	From 3 to 20	From 8 to 16
**Laboratory parameters**								
-Number studied in the study	9	3	7	16	9	4	12	10
-Measurement number per patient * (one/several/both)	NR	NR	Both	Several	Several	One	One	One
-Statistical analysis** (one/several/both)	NR	NR	Both	Several	Several	One	One	One

SCD: Sickle cell Disease, CV: Cerebral vasculopathy, NA: Not appropriated, NR: Not reported, PAH: Pulmonary arterial hypertension, VOC: vaso-occlusive crisis; *One means that laboratory parameter was collected once during the entire follow-up, several means that they were collected several times during the entire follow-up and both means that the measurement number varies according to the laboratory parameter within the same study; ** One means that only one measurement was taken into account in the statistical analysis, several means that several measurements were taken into account in the statistical analysis and both means that the statistical analysis varies according to the laboratory parameter within the same study.

References	[9]	[10]	[11]	[12]	[13]	[14]	[15]	[16]
Publication year	2013	2014	2014	2016	2016	2016	2016	2017
**Study**								
-Region	North America	North America	North America	Europe	North America	Europe	North America	Europe
-SCD event studied	Retinopathy	CV Mortality Stroke	Growth	Reverse CV	CV	CV	Silent cerebral infarction	Alloimmunization
**Population**								
-Inclusion number	123	354	193	46	121	375	50	175
-SCD genotype	SS/Sβ°	SS	SS/Sβ°	NR	NR	SS/Sβ°	SS/Sβ°	SS/Sβ°/SC
-Inclusion age (years)	13 (median)	4.8 (means)	13.6 (means)	From 0.5 to 3.1	From 2 to 15	From 0 to 1	9.4 (median)	11.9 (median)
**Laboratory parameter**								
- Number studied in the study	9	3	4	10	3	8	8	1
-Measure number per p tient* (one/several/both)	Several	One	One	Several	One	Several	One	NR
-Statistical analysis** (one/several/both)	NA	One	One	Several	One	Several	One	One

SCD: Sickle cell Disease, CV: Cerebral vasculopathy, NA: Not appropriated, NR: Not reported, PAH: Pulmonary arterial hypertension, VOC: vaso-occlusive crisis; *One means that laboratory parameter was collected once time during the entire follow-up, several means that they were collected several times during the entire follow-up and both means that the measurement number varies according to the laboratory parameter into the same study; ** One means that only one measurement was taking into account in the statistical analysis, several means that several measurements were taking into account in the statistical analysis and both means that the statistical analysis varies according to the laboratory parameter into the same study.

Bibliography

1. Bernaudin F, Verlhac S, Chevret S, Torres M, Coic L, Arnaud C, et al. G6PD deficiency, absence of alpha-thalassemia, and hemolytic rate at baseline are significant independent risk factors for abnormally high cerebral velocities in patients with sickle cell anemia. Blood. 2008;112:4314–7.
2. Hankins JS, Fortner GL, McCarville MB, Smeltzer MP, Wang WC, Li C-S, et al. The natural history of conditional transcranial Doppler flow velocities in children with sickle cell anaemia. Br J Haematol. 2008;142:94–9.
3. Kwiatkowski JL, Zimmerman RA, Pollock AN, Seto W, Smith-Whitley K, Shults J, et al. Silent infarcts in young children with sickle cell disease. Br J Haematol. 2009;146:300–5.
4. Colombatti R, Maschietto N, Varotto E, Grison A, Grazzina N, Meneghello L, et al. Pulmonary hypertension in sickle cell disease children under 10 years of age. Br J Haematol. 2010;150:601–9.
5. Bernaudin F, Verlhac S, Arnaud C, Kamdem A, Chevret S, Hau I, et al. Impact of early transcranial Doppler screening and intensive therapy on cerebral vasculopathy outcome in a newborn sickle cell anemia cohort. Blood. 2011;117:1130–40; quiz 1436.
6. Cox SE, Makani J, Fulford AJ, Komba AN, Soka D, Williams TN, et al. Nutritional status, hospitalization and mortality among patients with sickle cell anemia in Tanzania. Haematologica. 2011;96:948–53.
7. Darbari DS, Onyekwere O, Nouraie M, Minniti CP, Luchtman-Jones L, Rana S, et al. Markers of severe vaso-occlusive painful episode frequency in children and adolescents with sickle cell anemia. J Pediatr. 2012;160:286–90.
8. Lamarre Y, Romana M, Waltz X, Lalanne-Mistrih M-L, Tressières B, Divialle-Doumdo L, et al. Hemorheological risk factors of acute chest syndrome and painful vaso-occlusive crisis in children with sickle cell disease. Haematologica. 2012;97:1641–7.
9. Estepp JH, Smeltzer MP, Wang WC, Hoehn ME, Hankins JS, Aygun B. Protection from sickle cell retinopathy is associated with elevated HbF levels and hydroxycarbamide use in children. Br J Haematol. 2013;161:402–5.
10. Meier ER, Wright EC, Miller JL. Reticulocytosis and anemia are associated with an increased risk of death and stroke in the newborn cohort of the Cooperative Study of Sickle Cell Disease. Am J Hematol. 2014;89:904–6.
11. Rana S, Houston PE, Wang WC, Iyer RV, Goldsmith J, Casella JF, et al. Hydroxyurea and growth in young children with sickle cell disease. Pediatrics. 2014;134:465–72.
12. Bernaudin F, Verlhac S, Arnaud C, Kamdem A, Hau I, Leveillé E, et al. Long-term treatment follow-up of children with sickle cell disease monitored with abnormal transcranial Doppler velocities. Blood. 2016;127:1814–22.
13. Meier ER, Fasano RM, Estrada M, He J, Luban NLC, McCarter R. Early Reticulocytosis and Anemia Are Associated with Abnormal and Conditional Transcranial Doppler Velocities in Children with Sickle Cell Anemia. J Pediatr. 2016;169:227-231.e1.
14. Sommet J, Alberti C, Couque N, Verlhac S, Haouari Z, Mohamed D, et al. Clinical and haematological risk factors for cerebral macrovasculopathy in a sickle cell disease newborn cohort: a prospective study. Br J Haematol. 2016;172:966–77.
15. Nottage KA, Ware RE, Aygun B, Smeltzer M, Kang G, Moen J, et al. Hydroxycarbamide treatment and brain MRI/MRA findings in children with sickle cell anaemia. Br J Haematol. 2016;175:331–8.
16. Allali S, Peyrard T, Amiranoff D, Cohen JF, Chalumeau M, Brousse V, et al. Prevalence and risk factors for red blood cell alloimmunization in 175 children with sickle cell disease in a French university hospital reference centre. Br J Haematol. 2017;177:641–7.
